# Response to Peter Mantle. Comments on “Mycobiota and Mycotoxins in Traditional Medicinal Seeds from China. *Toxins* 2015, 7, 3858-3875”—Rigour in Attributing Ochratoxin A Biosynthesis within the Genus *Penicillium* Occurring on Natural Agricultural Produce

**DOI:** 10.3390/toxins8060167

**Published:** 2016-05-31

**Authors:** Weiwei Gao

**Affiliations:** Institute of Medicinal Plant Development, Chinese Academy of Medical Sciences and Peking Union Medical College, Beijing 100193, China; wwgao411@sina.com; Tel.: +86-10-5783-3423; Fax: +86-10-5783-3020

My colleagues and I appreciate the comments and constructive suggestions on our manuscript “Mycobiota and Mycotoxins in Traditional Medicinal Seeds from China”. We used the direct culture method and dilution plate method to isolate internal and superficial contaminating fungi from seed samples. Seven *Penicillium polonicum* isolates were obtained from the surface of tangerine and lychee seeds; only two of which were identified as a potential source of ochratoxin A (OTA) contamination. The comment suggested that mycotoxin occurrence should be detected in the single-spore isolates of two isolates. Although the dilution plate method-obtained isolates are usually original from single-spores, there is still a possibility that they could be contaminated by other fungi. So we accept the comment and will use monoconidial cultures of the two isolates of *P. polonicum* for OTA detection in our future work.

The comment also mentioned *P. polonicum* cannot produce OTA according to experience in Europe. However, our laboratory confirmed the ochratoxigenic capacity of this species by using various strains isolated from different host plants (tangerine seed, lychee seed, fresh and dry liquorice root [1,2]) through independent fungal cultures and by using different determination methods (UPLC-MS/MS and LC-MS/MS [1,2]). Therefore, there might be ochratoxigenic potential in certain *P. polonicum* strains. The toxigenic feature of this species therefore needs to be confirmed in further studies.
